# Amyloid Beta Peptides and Th1 Cytokines Modulate Human Brain Vascular Smooth Muscle Tonic Contractile Capacity In Vitro: Relevance to Alzheimer’s Disease?

**DOI:** 10.3390/pathophysiology28010006

**Published:** 2021-02-11

**Authors:** J. Winny Yun, Caretia Washington, Joi McCormick, Emily Stevenson, J. Steven Alexander

**Affiliations:** Department of Molecular and Cellular Physiology, LSUHSC, Shreveport, LA 71103, USA; jyun@lsuhsc.edu (J.W.Y.); caretia.washingt@ufl.edu (C.W.); joimccormick12@gmail.com (J.M.); emily.v.stevenson@gmail.com (E.S.)

**Keywords:** Alzheimer’s disease, inflammatory cytokine, vascular smooth muscle, tonic contraction

## Abstract

Alzheimer’s Disease (AD) is a neurodegenerative condition characterized both by the presence of tau protein neurofibrillary tangles and amyloid beta (Aβ) containing extracellular “plaques”. The cleavage of amyloid precursor protein (APP) yields several Aβ peptides. Although Aβ toxicity to neurons has been described extensively, its effects on other components of the neurovasculature such as vascular smooth muscle cells have been less well characterized. AD is now also recognized as a neurovascular disease characterized by cerebral microbleeds and disturbances in autoregulation. AD is also a neuroinflammatory condition in which several proinflammatory cytokines are elevated and may contribute to the intensification of AD severity. Cerebral autoregulation (the mechanism by which brain blood flow is maintained despite changes in perfusion pressure) is extremely tightly controlled in the brain and shows disturbances in AD. The failure of autoregulation in AD may make the brain susceptible to cerebral microbleeds through a reduced capacity to limit blood flow when pressure is increased. Conversely, reduced vasodilation during low flow might could also exacerbate tissue hypoxia. Currently, whether and how Aβ peptides and inflammatory cytokines depress brain smooth muscle cell tonic contraction is not known, but could reveal important targets in the preservation of autoregulation which is disturbed in AD. We used a collagen gel contractility assay to evaluate the influence of Aβ25-35, Aβ1-40 and Aβ1-42 peptides and inflammatory cytokines on the tonic contractility of human brain vascular smooth muscle cells (HBVSMC) as an in vitro model of cerebral autoregulation. We found that 5 and 10 μM Aβ1-42 significantly depressed HBVSM contractility, while Aβ1-40 5–20 μM had no effect on contractility. Conversely, Aβ25-35 (1–50 μM) increased contractility. Interestingly, the inflammatory cytokines TNF-α (20 ng/mL), IL-1β (20 ng/mL) and IFN-γ (1000 U/mL) also depressed HBVSM tonic contractility alone and in combination. These data suggest that both the inflammatory milieu in AD as well as the abundance of Aβ peptides may promote autoregulatory failure and increase brain susceptibility to dysregulated perfusion and microbleeds which are an important and devastating characteristic of AD.

## 1. Introduction

Alzheimer’s disease (AD) is a progressive neurodegenerative disease affecting more than 5 million individuals in the United States and 17 million worldwide [1,2]. AD is characterized by progressive cognitive decline, which appears secondary to the intracellular accumulation of fibrillary tangles composed of hyper-phosphorylated tau and extracellular accumulation of amyloid beta (Aβ)-containing plaques in the brain [3]. Several forms of Aβ are produced by a two-step enzymatic cleavage of the amyloid precursor protein (APP), a transmembrane protein abundant in neurons [4] and other cells, including vascular endothelial cells [5]. Depending on the enzymes involved in APP processing, APP can be cleaved to produce several toxic forms of Aβ (when cleaved sequentially by β- and α-secretase) or non-amyloidogenic sAPPα (when cleaved by alpha-secretase and other proteases)—these events have been extensively reviewed [6,7,8]. Following the amyloidogenic cleavage of APP, Aβ monomers spontaneously assemble into oligomers that eventually form insoluble proteostatic Aβ plaques, which represent key mediators of AD pathology [9].

The presence of tau and Aβ accumulation in AD brains is by now well characterized, and extensive in vivo and in vitro studies have shown the disastrous effects of the over-abundance of these proteins in neuronal signaling, biological function and survival [10,11]. However, AD research no longer considers neurons in isolation but has broadened to include the interactions within neurovascular unit [12]. In fact, several lines of evidence have shown that risk factors for cardiovascular disease can also exacerbate the development of dementia and AD [13]. For example, diabetes and metabolic syndrome are significant risk factors that hasten and intensify both human and experimental models of AD [14,15] through activation of vascular inflammatory programs. Further, cerebrovascular dysfunction has been shown to occur in AD long before the onset of AD symptoms [16]. Changes in cerebral blood flow have been implicated as a strong predictive factor in AD [12,17,18]. A novel model of decreased cerebral blood flow in mice recently demonstrated the increased production and deposition of Aβ on cerebral blood vessels and in the brain [19], suggesting that cerebrovascular disturbances play an important role in the progression of AD. However, the mechanisms for how this may occur and the precise contribution of the interaction between the endothelium and smooth muscle of the cerebrovasculature in the progression of AD have not yet been determined.

In addition to the toxic effects of Aβ peptides on the neurovasculature, AD is increasingly recognized as a neurovascular inflammatory disease. Pro-inflammatory cytokines have been implicated in regulating APP processing and modulating Aβ levels [1], and inflammatory markers are present in the AD afflicted brain [20,21]. Such cytokines include IL-1β, TNF-α and IFN-γ [2]. However, their effects on brain vascular smooth muscle cells and cerebral autoregulation has not been extensively studied.

Cerebral autoregulation, the ability of brain vascular smooth muscle cells to acutely contract and dilate within seconds of changes in the perfusion pressure to stabilize blood flow [22] is a critical function of the neurovasculature. Consequently, disturbances in cerebral smooth muscle contractile capacity might impair cerebral autoregulation, a vascular manifestation of AD [23]. In addition to modulating autoregulation, brain vascular smooth muscle cells are also recognized as an important component in regulating brain lymphatic drainage. Impaired brain vascular smooth muscle contractility is associated with decreased lymphatic drainage and clearance of toxins in the brain, including Aβ [24,25].

Here, we examined the effects of Aβ peptides as well as inflammatory cytokines on the tonic contraction of brain vascular smooth muscle cells, which are responsible for controlling cerebral autoregulation and Aβ clearance from the brain. We report here the differential effects of Aβ peptides on human brain vascular smooth muscle cells (HBVSMC) tonic contractility and also demonstrate that inflammatory cytokines significantly depressed human brain vascular smooth muscle contractility. We propose that the increased abundance of Aβ peptides and elevated inflammatory cytokines in AD could potentially dysregulate cerebral vasomotion, which might impair cerebral autoregulation seen in this condition.

## 2. Materials and Methods

### 2.1. Cell Culture

Human brain vascular smooth muscle cells (HBVSMC) were purchased from ScienceCell and cultured in Smooth muscle cell medium (SMCM) (ScienceCell, Carlsbad, CA, USA) supplemented with 10% FBS (Atlanta Biologicals, Flowery Branch, GA, USA), 1% penicillin-streptomycin-amphotericin B (PSA, Coring Cellgro, Herndon, VA, USA) and 200 μM glutamine (HyClone Laboratories, Logan, UT, USA). Cells were cultured at 37 °C in 7.5% CO_2_. Cell culture medium was changed weekly and cells split every 14 days at a 1:5 ratio.

### 2.2. Collagen Gel Contraction Assay

#### 2.2.1. Preparation of Rat Tail Type 1 Collagen

Rat tail type 1 collagen matrices were prepared by a modification of the protocol previously published by Benoit et al. (2008). Briefly, rat tail tendons were manually excised, washed with 100% isopropanol (ThermoFisher, Waltham, MA, USA) and dissolved in sterile 4 mM acetic acid for 24 hours at 4 °C under constant agitation. Collagen solution was filtered through a 250 μm nylon filter (Spectrum Labs, Rancho Dominguez, CA, USA), centrifuged at 19× *g* for 20 min at 4 °C and snap frozen. Using a bench-top manifold freeze-dryer (Millrock Technology, Kingston, NY, USA), frozen aliquots were dried and stored at −20 °C for future use.

#### 2.2.2. Preparation of HBVSMC/Collagen Gel

Twenty-four hours prior to experiments, freeze-dried collagen was solubilized in cold 0.012 M hydrochloric acid (HCl) (Sigma-Aldrich, St. Louis, MO, USA) at 2.5 mg·mL^−1^ final collagen concentration and incubated overnight at 4 °C with gentle agitation. On the day of the experiment, 0.8 mL of cold 5× PBS was added to 3.2 mL of dissolved collagen gel and the pH was titrated with 0.5 M sodium hydroxide (NaOH) (Sigma-Aldrich, St. Louis, MO, USA) to 7.4. Cultured HBVSMC were washed twice with PBS and then harvested with trypsin-EDTA (Sigma-Aldrich, St. Louis, MO, USA). These cells were centrifuged at 485× *g* for 5 min, resuspended in DMEM (supplemented with 10% FBS, PSA and glutamine) and counted. A total of 1.2 × 106 cells (50,000 cells per well) were resuspended in 8 mL of supplemented DMEM. The final HBVSMC/collagen mixture (8 mL of cell suspension in 4 mL of collagen gel solution) was seeded in 500 μL aliquots into 24-well plates (ThermoFisher, Waltham, MA, USA) and incubated at 37 °C for 1 h to polymerize. In our tonic contraction, assay gels maintained established levels of tonic contractility and did not exhibit relaxation or “fatigue” once contracted.

### 2.3. Collagen Gel Treatment with Aβ and Cytokines

HBVSMC incorporated into collagen gels were prepared in 1 mL DMEM (supplemented with 10% FBS, PSA and glutamine) supplemented to a final concentration with Aβ (1, 5, 10, 50 μM) or cytokines: h-IL-1β (20 ng/mL), h-TNF-α (20 ng/mL), h-IFN-γ (400, 1000 U·mL^−1^), optimized in a dose–response assay previously [26]. Gels were then gently detached from the edges of the plates to allow unimpeded contraction and incubated for 4 days at 37 °C in 7.5% CO_2_. In these experiments, HBVSMC incorporated into gels attach to collagen fibers and promote progressive gel contraction over time. To monitor time and treatment-dependent changes in contraction, digital photographs of gels were recorded daily over 7 days (Nikon D40, Tokyo, Japan). Gel contractions in four replicate wells (quadruplicates) were averaged for each single *n* value, with *n* = 3 per group (i.e., 12 gels analyzed for *n* = 3).

### 2.4. Gel Contraction Analysis

Gel contraction was defined as the change in gel surface area on day 3 or 4 as a fraction of its area measured on day 0, and normalized to internal controls. All measurements were made using the NIH ImageJ analysis program (Schneider et al., 2012). At day 0, gel surface areas were initially equal to the well surface area, as immediately after gel polymerization contraction had not yet begun. Over 4 days, HBVSMC contraction reduced the respective gel area, which was found to show the greatest differences at this time point. On day 3 or 4, gel surface areas were measured, and area changes were determined as the gel area divided by the initial gel surface area on day 0. This value was subtracted from 1 to express it as a fractional change in area and then normalized to the contraction in control gels within each experiment, which was set as 100% contraction. This normalization to internal control was critical due to variations between batches of prepared HBVSMC–collagen samples.

### 2.5. Data Analysis

Data are presented as mean ± SEM. When comparing three or more experimental groups, one-way ANOVA with Dunnett’s post hoc testing was used. Differences between two sets of data were determined using the unpaired two-tailed Student’s *t*-test (GraphPad Prism software, San Diego, CA, USA, version 6). Comparisons were considered statistically significant at *p* < 0.05.

## 3. Results

### 3.1. Aβ Peptides Disturb HBVSMC Contractility

**Figure 1:**
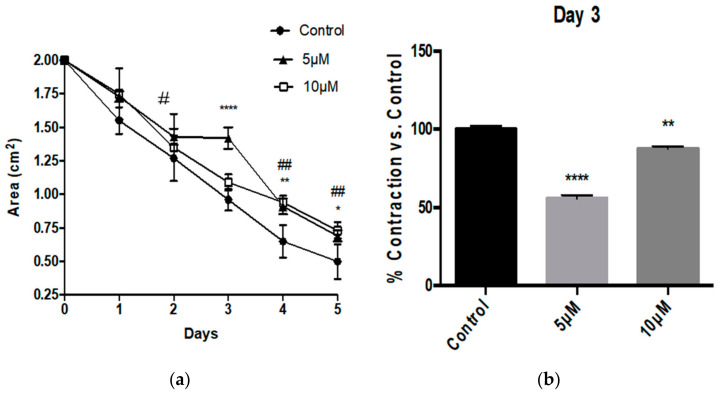

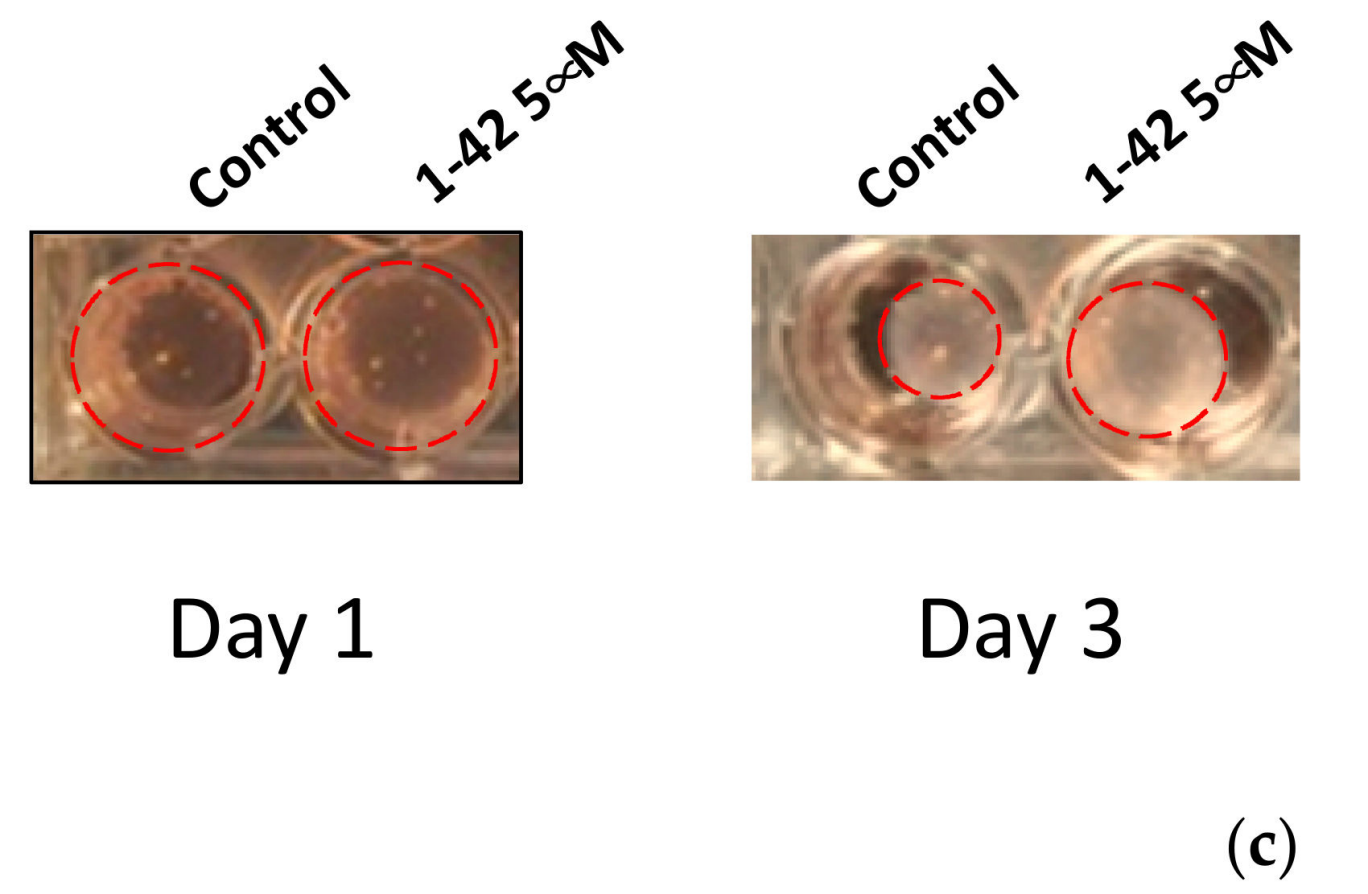
Aβ1-42 peptide decreased HBVSMC contractility. (**a**) Human brain vascular smooth muscle cells (HBVSMC) treated with 5 or 10 μM of Aβ1-42 exhibit significantly larger area after 3 days of treatment (days 3–5). (**b**) Aβ1-42 decreased HBVSMC contractility at day 3 significantly compared with control *n* = 4, * *p* < 0.05, ** *p* < 0.01, **** *p* < 0.001 for 5 uM treatment vs. control on each day, # *p* < 0.05, ## *p* < 0.01 for 10 uM treatment vs. control each day; one-way ANOVA with Dunnett’s post-testing; data are mean ± SEM. (**c**) Representative figure of HBVSMC/collagen gels incubated with or without 5 μM of Aβ1-42 peptides over the period of 3 days. Red dashed lines indicate the outline of the HBVSMC/collagen gels which were used to calculate the area.

HBVSMC were stimulated with Aβ1-42, Aβ1-40 and Aβ25-35 peptides. Aβ1-42 treatment resulted in a greater area of collagen gels over the course of 5 days starting on day 3 of treatment (Figure 1a). This translates to significantly depressed tonic contractility expressed as fractional contraction compared to control HBVSMC that received no Aβ1-42 treatment. Both 5 and 10 μM Aβ1-42 depressed tonic contractility on days 3, 4 and 5, with the most prominent effect observed on day 3 (Figure 1b).

**Figure 2:**
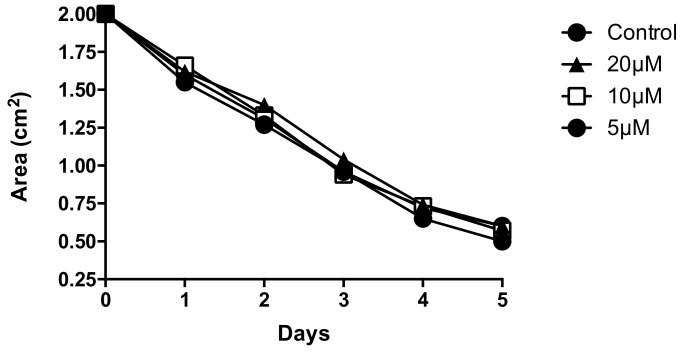
Aβ1-40 peptides did not alter HBVSMC contractility. At 5, 10 or 20 μM. Aβ1-40 did not significantly affect HBVSMC contractility at 5, 10 or 20 μM over 5 days. *n* = 4, data are mean ± SEM.

**Figure 3:**
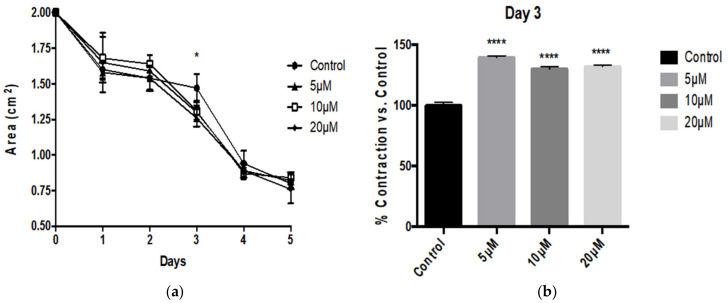
Aβ25-35 peptide enhanced HBVSMC contractility. (**a**) HBVSMC in collagen gel treated with various concentrations of Aβ25-35 exhibit a smaller area at 3 days of treatment. This effect was not observed after 4 days of treatment. (**b**) At 3 days in culture, Aβ25-35 treatment significantly enhanced HBVSMC contractility at all concentrations tested, from 1–50 μM compared with control *n* = 4, * *p* < 0.05, **** *p*<0.0001 versus control; one-way ANOVA with Dunnett’s post-testing; data shown are mean ± SEM.

Paradoxically we found that Aβ1-40 did not significantly affect tonic contractility of HBVSMC in collagen gel over 5 days (Figure 2). Interestingly however, we found that the smaller fragment Aβ25-35 enhanced tonic contractility of HBVSMC (Figure 3). Particularly on day 3 we observed a dramatic and significant increase in contractility at all concentrations tested (Figure 3b).

### 3.2. Inflammatory Cytokines Suppress Human Vascular Smooth Muscle Tonic Contractility

**Figure 4:**
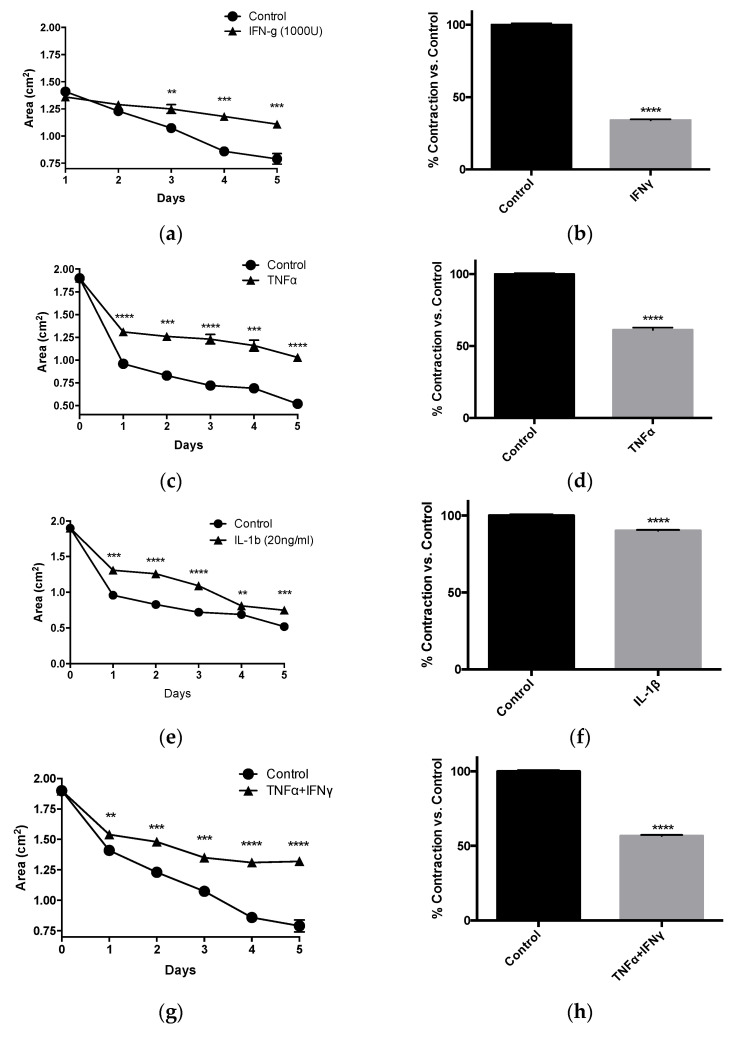

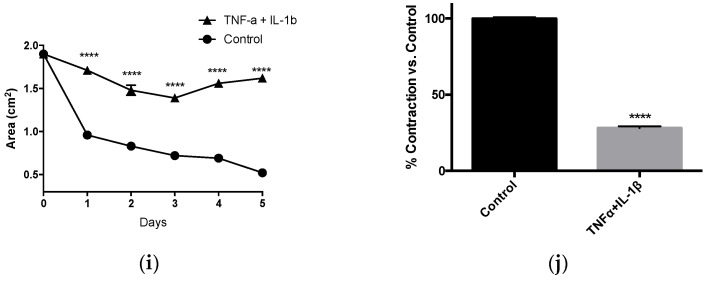
Inflammatory cytokines depress HBVSMC contractility. (**a**) 1000 U/mL of IFNγ treatment on HBVSMC resulted in greater area (measured in cm^2^) after 3 days. (**b**) HBVSMC treated with 1000 U/mL of IFNγ for 4 days showed a significantly lower contractility (expressed in % of contraction vs. control) compared with control treated HBVSMC. (**c**) 20 ng/mL of TNFα treatment on HBVSMC resulted in greater areas after 1 day up to 5 days. (**d**) HBVSMC treated with TNFα for 4 days showed a significantly lower contractility compared to control HBVSMC. (**e**) 20 ng/mL of IL-1b treatment on HBVSMC in collagen gel resulted in a larger area after 1 day up to 5 days. (**f**) HBVSMC treated with 20 ng/mL of IL-1b for 4 days showed a significantly lower contractility compared to control HBVSMC. (**g**) A combined treatment consisting of 1000 U/mL of IFNγ and 20 ng/mL of TNFα on HBVSMC in collagen gel resulted in even greater areas (measured in cm^2^) after 1 day. This effect persisted throughout the study (5 days). (**h**) HBVSMC treated with a combination of 1000 U/mL of IFNγ and 20 ng/mL of TNFα for 4 days showed a significantly lower contractility. (**i**) A combined treatment consisting of 20 ng/mL of IL-1b and 20 ng/mL of TNFα on HBVSMC in collagen gel resulted in a larger area (measured in cm^2^) after 1 day. This effect persisted throughout the study (5 days). (**j**) HBVSMC treated with a combination of 20 ng/mL of IL-1b and 20 ng/mL of TNFα for 4 days showed a significantly lower contractility. *n* = 3, ** *p* < 0.01, *** *p* < 0.001, **** *p* < 0.0001 versus control; Student’s *t*-test; data are mean ± SEM.

Aβ can promote microglial activation resulting in elevated production of inflammatory cytokines, which can in turn damage the neurons to aggravate AD pathologies [27]. However the effect of these inflammatory cytokines on the tonic contractility of HBVSMC has not been studied. We analyzed the direct effect of recombinant TNF-α (20 ng/mL), IL-1β (20 ng/mL) and IFN-γ (1000 U/mL) on HBVSMC contractility. As shown in Figure 4, treatment with IFN-γ (Figure 4a,b), TNF-α (Figure 4c,d) or IL-1β (Figure 4e,f) alone significantly depressed the tonic contractility of HBVSMC expressed as fractional contractility compared to the control (no treatment), as well as when expressed as an area of the collagen gel (cm^2^). Combination treatment of IFN-γ with TNF-α (Figure 4g,h) or TNF-α with IL-1β (Figure 4i,j) demonstrated a greater impairment of contraction compared to singular cytokine treatments.

## 4. Discussion

AD, the most common forms of age-related dementia, is commonly characterized by the presence and accumulation of Aβ containing plaques that result from proteolytic cleavage of the precursor APP by β-and γ- secretase instead of the physiological α-secretase [3,4]. Following the cleavage by β-secretase, another enzymatic cleavage occurs by γ-secretase. The variable nature of this amyloid processing results in C-terminal heterogeneity yielding several Aβ products. Of the various Aβ species that result from the imprecise cleavage by β-secretase, the most abundant form is Aβ1-40, which can make up approximately 80–90%, followed by Aβ1-42, which make up 5–10% of the total amyloid generated [5]. The longer forms (particularly Aβ1-42) are more hydrophobic with the highest tendency to form aggregates. Aβ1-42 is considered to be the main pathogenic form of amyloid deposited in the brain in AD, [5,6]. The shorter Aβ25-35 peptide is produced from proteolytic cleavage of Aβ1-40 [7]. This form is the shortest fragment studied to date that exhibits the ability to form β-sheet aggregated structures and retains the toxicity of the full-length peptide [8].

Cerebral autoregulation is dictated by the ability of cerebral vascular smooth muscle cells to contract and dilate within seconds of changes in the perfusion pressure to stabilize blood flow [22]. A dysfunction in this mechanism can be associated with hypoperfusion of the brain on one end of the spectrum and microbleeds on the other, contributing to the vascular contributions in AD [23]. Prevalence of microbleeds in AD patients is reported to be 23%, and among patients with microbleeds 50% have experienced only one micro-bleed, while the other 50% experienced at least two events [24].

In our study we found that Aβ1-42 peptides depressed HBVSMC contractility as early as 3 days after treatment, with resulting contractility of 80.74% with 5 μM and 78.51% with 10 μM. This decrease in contractility was sustained throughout the experiment until day 5 of treatment at both concentrations tested. On day 4 after treatment the resulting area for control HBVSMC-collagen gel was 0.65 ± 0.12 cm^2^, which is still dramatically and significantly smaller compared to the area displayed by HBVSMC–collagen samples that received 5 μM (0.91 ± 0.06 cm^2^) or 10 μM (0.94 ± 0.05 cm^2^) of Aβ1-42 peptides. Although contractility appears to be significantly depressed on all later days of the experiment (days 3, 4 and 5), the greatest effect on contractility was observed on day 3, and the amplitude of the effect decreased on days 4 and 5. These results appear consistent with previous reports by Hald et al. in which Aβ1-42 isoforms were found to reduce vascular smooth muscle contractile output and in turn decrease vasodilatory capacity of cerebrovascular vessels [26]. Conversely, Aβ25-35 enhanced the tonic contraction of HBVSMC, at day 3 of treatment. However this effect, unlike the depression of contractility resulting from Aβ1-42, was transient and could not be measured after 4 days of treatment. Nonetheless Aβ25-35 peptide treatment did not cause the impairment of contraction as observed following the full-length (Aβ1-42) treatment. This indicates that although Aβ25-35 is known retain the toxicity of the full-length peptides, it does not retain its effect on HBVSMC contraction. Interestingly, Aβ1-40 had no significant effect on the tonic contractility of HBVSMC and no difference compared to the control-treated cells were observed— once again, not retaining the same effect as Aβ1-42.

**Figure 5:**
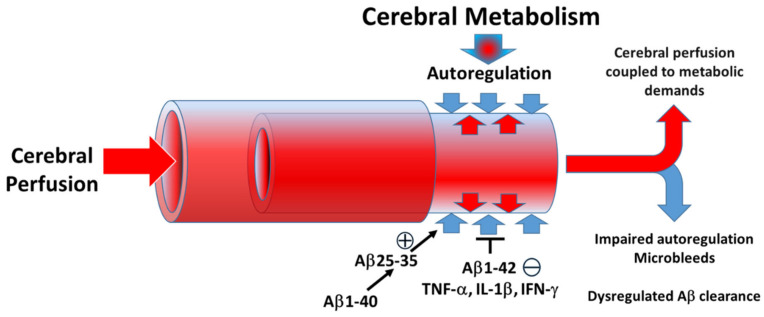
Disturbances in autoregulation produced by Aβ1-42 and inflammatory cytokines (TNF-α, IL-1β and IFN-γ) could lead to impaired (−) cerebral perfusion and contribute to microbleeds and dysregulated Aβ clearance. Aβ25-35 may enhance constriction (+). We were unable to observe effects of Aβ1-40 on human brain smooth muscle contractility.

AD pathology, although most commonly associated with Aβ plaques, is also intimately associated with inflammation and the production of inflammatory mediators, especially pro-inflammatory cytokines [9,10]. Increasing evidence also implicates neurovascular inflammatory disturbances in the progression of Alzheimer’s disease [11,12]. In particular, IL-1β levels have been shown to correlate with changes in the expression of APP isoforms; TNF-α has also been identified as a key mediator that stimulates BACE1 expression to enhance amyloidogenic processing of APP, in addition to inducing APP mRNA expression [13]. IFN-γ has also been reported to be upregulated in AD brains and could enhance production of Aβ production as well as deposition [2,14]. Although microglia and neurons represent important potential targets of cytokine and amyloid peptides in AD [13,15], far fewer studies have considered the effects of these mediators on brain vascular smooth muscle contractile capacity [1,2]. Our data now suggest that inflammatory cytokines also directly affect brain vascular smooth muscle cells responsible for maintenance of constant blood flow via cerebral autoregulation (Figure 5).

Contractility was assessed following treatment with cytokines which are known to be important players in AD [27]. Cytokine treatments were carried out both individually and also in combination in order to evaluate the effect of these cytokines alone and simultaneously. All of the pro-inflammatory cytokines tested here depressed smooth muscle cell tonic contraction. Specifically, we found that with individual treatments, IFN-γ most potently depressed contractility of HBVSMC (33% vs. control) followed by TNF-α (61%) and IL-1β (90%). Interestingly IL-1β combined with TNF-α elicited an amplified effect, impairing contraction to 28% compared to control samples. IFN-γ combined with TNF-α depressed contractility to 56%. Smooth muscle cell contractility is critical for sustained resistance against increases in feeding pressures. Impaired smooth muscle cell contractility is strongly associated with microbleeds, which is not uncommon with AD patients [16]. AD patients with multiple microbleeds have been shown to have more severely impaired cognition compared with patients without any brain microbleeds [17], implicating microbleeds in AD disease progression.

Additionally, we have previously reported the effects of pro-inflammatory cytokines on the brain endothelial cells that can alter the tonic contraction of brain vascular smooth muscle cells. Treatment with pro-inflammatory cytokines, such as TNF-α and IFN-γ, enhances the basolateral release of microparticles (MPs), which are defined as extracellular vesicles between 0.1 and 1 μm that form by direct budding of the plasma membrane [18]. The basolateral MPs (BMPs) released from healthy brain endothelial cells enhanced HBVSMC contractility; however, BMPs collected from cells treated with pro-inflammatory cytokines significantly depressed contractility. In these experiments no cytokines were directly added to the HBVSMC indicating yet another pathway by which inflammatory cytokines can influence the tonic contraction of vascular smooth muscle cells via the interaction of the brain endothelium and smooth muscle.

In addition to autoregulation, brain vascular smooth muscle contractility is also recognized to be important in brain lymphatic drainage and clearance of toxins, including Aβ [19]. Impaired clearance can effectively lead to the buildup of Aβ deposition leading to increased plaque burden and AD. In this study we demonstrated the differential effects of Aβ peptides and pro-inflammatory cytokines on the tonic contractility of HBVSMC that may exacerbate conditions such as AD. Thus, pro-inflammatory cytokines are elevated in AD as well as Aβ peptides; Aβ1-42 in particular may play an important role in depressing HBVSMC contractile capacity, impairing clearance as well as autoregulation in the brain.

Aβ25-35, the smallest, toxic form of Aβ which can self-assemble, also induces neuroinflammation in astrocytes and microglia associated with disturbances in memory consolidation [20]. Aβ25-35 is also directly toxic to endothelial cells in culture [21], and Mukhamediarov et al. [22] found that Aβ25-35-peptides depolarize skeletal muscle (in mice). Aβ25-35-peptides may also dysregulate calcium handling in cardiomyocytes leading to arrhythmias. Similarly, Haase et al. [23] also showed that Aβ25-35 also activates α1 adrenergic receptors, which in smooth muscle are known to enhance contractility.

Melchor et al. [24] have observed that Aβ peptides and APP accumulate in human cerebrovascular smooth muscle cells: effects which may contribute to AD pathophysiology. Coma et al. [25] reported that vascular smooth muscle cells in culture have all the necessary machinery to process APP and generate Aβ1-42 and Aβ1-40 peptides. That study also found that oxidant stress increased beta-amyloid converting enzyme 1 (BACE1) expression which could promote Aβ formation by vascular smooth muscle; other secretases (e.g., γ-secretase) were not enhanced. Therefore, cerebral amyloid angiopathy pathology in AD could be vascular as well as smooth muscular in its origins.

Like Aβ25-35, Aβ1-40 is also stressful to endothelial and smooth muscle cells with Aβ1-40 even being lethal to vascular smooth muscle [28]. Interestingly, changes in smooth muscular vascular reactivity may be endothelial-dependent. However, these pathologic smooth muscle–endothelial interactions may be independent of matrix metalloprotease activity.

Conversely, Aβ1-42 appears to be directly toxic to endothelial cells [29] and cerebrovascular smooth muscle cells [29]. Aβ1-42 promotes degeneration of smooth muscle cells [30]—an effect that was prevented by inhibition of nephrilysin in smooth muscle. Similarly, human brain pericytes, which are structurally and functionally similar to brain vascular smooth muscle, and leptomeningeal smooth muscle cells were also degenerated by wild-type Aβ1-42, while wild-type Aβ1-40 was inactive. Interestingly, that study showed that “Dutch” mutations in these proteins had different activities with “Dutch” Aβ1-42 (HCHWA-D) being inactive, but “Dutch” Aβ1-40 was able to induce degeneration in these cells [31].

Here, we found that Aβ25-35 enhanced, while Aβ1-42 (but not Aβ1-40) depressed contractility in human brain vascular smooth muscle cells. These data appear consistent with these previously reported results. Models which incorporate endothelial cells and other components of the neurovasculature (neurons, astrocytes) may be able to even more fully recapitulate these responses to Aβ peptides as they relate to AD. Additionally, while it is difficult to directly compare the effects of cytokines and amyloid peptides, HBVSMC contractility may be more sensitive to cytokines. One possible explanation is due to the presence of specific cytokine receptors on smooth muscle that may make these cells highly and selectively sensitive to cytokine signals. By comparison, amyloid peptide may provoke stress by binding to many Ab “receptors” that are not specific or selective and often inconsistently demonstrated on smooth muscle (LRP-1, a1-adrenergic receptors, TLR2, NMDA and AMPA receptors).

In summary, we have demonstrated that different types of Aβ peptides produce differential effects on HBVSMC tonic contractility, with Aβ1-42 showing a sustained depression of HBVSMC contractility, while Aβ25-35 may transiently enhance HBVSMC contractility. Pro-inflammatory cytokines associated with AD also significantly and dramatically depressed HBVSMC contractility. Combinations of these inflammatory and toxic species may influence the contractile capacity of human brain vascular smooth muscle which may contribute to impaired cerebral autoregulation and its sequelae in AD, as well dysregulating intramural periarterial drainage (IPAD) [32] of Aβ, reducing its clearance from the central nervous system. Therapies which may maintain normal cerebral vascular muscular functionality may be critical to maintaining normal brain blood pressure and distribution, as well as contributing to amyloid clearance in AD prevention and therapy.

## Data Availability

Not applicable.
